# Determinants of Job Satisfaction among Nurses from Chilean Hospitals

**DOI:** 10.17533/udea.iee.v41n3e04

**Published:** 2023-10-10

**Authors:** Marta Simonetti, Leyla Sáez

**Affiliations:** 1 Registered Nurse, PhD. Full Professor, School of Nursing, Universidad de los Andes, Chile. Email: msimonetti@uandes.cl School of Nursing Universidad de los Andes Chile msimonetti@uandes.cl; 2 Registered Nurse, Master’s. Assistant Professor, School of Nursing, Universidad Católica del Maule, Curicó-Chile. Email: lsaez@ucm.cl Universidad Católica del Maule School of Nursing Universidad Católica del Maule Curicó Chile lsaez@ucm.cl

**Keywords:** job satisfaction, nurses, hospitals, personnel turnover, working conditions, Chile, satisfacción en el trabajo, enfermeras y enfermeros, hospitales, reorganización del personal, condiciones de trabajo, Chile, satisfação no emprego, enfermeiras e enfermeiros, hospitais, reorganização de recursos humanos, condições de trabalho, Chile

## Abstract

**Objective.:**

To measure, at the national scope, the satisfaction of Chilean nurses working in hospitals, and establish personal and institutional determinants associated with satisfaction.

**Methods.:**

Cross-sectional multicenter study, carried out in 40 public and private high-complexity hospitals in Chile. A self-administered survey was conducted with 1,632 clinical nurses from medical-surgical units. The variables of interest studied were: job satisfaction, personal determinants (sex, age, and postgraduate training), institutional organizational determinants (assignments and work environment, measured through the *Practice Environment Scale of the Nursing Work Index)*, and institutional structural determinants. Data analysis applied hierarchical logistic regression models, with three blocks of determinants, following nested models design.

**Results.:**

The study showed that 21% of the nurses is very satisfied with their job. Training opportunities and professional growth are specific work aspects with which there is a lower percentage of nurses satisfied (10% and 11.2%, respectively). Among the personal factors, male sex and age are associated positively with satisfaction (*p*<0.05). Among the institutional organizational factors, a good work environment was associated with greater satisfaction (*p*<0.001); the number of patients per nurse was associated marginally with satisfaction (*p*<0.05). The structural factors of hospitals were not associated with satisfaction.

**Conclusion.:**

A low proportion of nurses working in the high-complexity hospitals studied are satisfied with their job. Planning of strategies must be prioritized, leading to improving the retention of nurses, reducing the number of patients per nurse, and promoting good work environments in hospitals.

## Resumen

**Objetivo.:**

Realizar una medición de alcance nacional de la satisfacción de las enfermeras chilenas que trabajan en hospitales para establecer qué determinantes personales e institucionales están asociados a ella.

**Métodos.:**

Estudio multicéntrico de corte transversal, realizado en 40 hospitales públicos y privados de alta complejidad en Chile. 1632 enfermeras clínicas de unidades médico-quirúrgicas diligenciaron una encuesta. Las variables de interés estudiadas fueron: satisfacción laboral, determinantes personales (sexo, edad y formación de postítulo), determinantes institucionales-organizacionales (dotaciones y ambiente laboral, medidos mediante el *Practice Environment Scale of the Nursing Work Index)* y determinantes institucionales estructurales. Para el análisis de los datos se aplicaron modelos de regresión logística jerárquica, con tres bloques de determinantes, con el diseño de modelos anidados.

**Resultados.:**

El 21% de las enfermeras estaba muy satisfecha con su trabajo. Las oportunidades de formación y de crecimiento profesional son los aspectos específicos del trabajo con los que hay menor porcentaje de enfermeras satisfechas (10% y 11.2%, respectivamente). Entre los factores personales, el sexo masculino y la edad se asocian positivamente a la satisfacción (*p*<0.05). Entre los factores institucionales organizacionales, un ambiente laboral bueno se asoció a mayor satisfacción (*p*<0.001); el número de pacientes por enfermera se asoció marginalmente a la satisfacción (*p*<0.05). Los factores estructurales de los hospitales no se relacionaron a la satisfacción.

**Conclusión.:**

Una baja proporción de enfermeras que trabaja en los hospitales de alta complejidad estudiados están satisfechas con su trabajo. Debe priorizarse la planificación de estrategias conducentes a mejorar la retención de las enfermeras, reducir el número de pacientes por enfermera y fomentar los buenos ambientes de trabajo en los hospitales.

## Resumo

**Objetivo.:**

Realizar uma medição nacional da satisfação dos enfermeiros chilenos que trabalham em hospitais e estabelecer quais determinantes pessoais e institucionais estão associados à satisfação.

**Métodos.:**

Estudo transversal multicêntrico, realizado em 40 hospitais públicos e privados de alta complexidade no Chile. 1632 enfermeiros clínicos de unidades médico-cirúrgicas responderam à pesquisa. As variáveis ​​de interesse estudadas foram: satisfação no trabalho, determinantes pessoais (sexo, idade e formação de pós-graduação), determinantes institucionais organizacionais (pessoal e ambiente de trabalho, mensurados por meio da Escala de Ambiente de Prática do Índice de Trabalho em Enfermagem) e determinantes institucionais estruturais. Para análise dos dados foram aplicados modelos de regressão logística hierárquica, com três blocos de determinantes, seguindo desenho de modelos aninhados.

**Resultados.:**

21% dos enfermeiros estavam muito satisfeitos com o seu trabalho. As oportunidades de formação e crescimento profissional são os aspectos específicos da função com os quais existe menor percentagem de enfermeiros satisfeitos (10% e 11.2%, respectivamente). Entre os fatores pessoais, o sexo masculino e a idade estão positivamente associados à satisfação (*p*<0.05). Dentre os fatores institucionais organizacionais, um bom ambiente de trabalho esteve associado à maior satisfação (*p*<0.001); O número de pacientes por enfermeiro esteve marginalmente associado à satisfação (*p*<0.05). Os fatores estruturais hospitalares não estiveram relacionados à satisfação.

**Conclusão.:**

Uma baixa proporção de enfermeiros que atuam nos hospitais de alta complexidade estudados estão satisfeitos com seu trabalho. Deve ser dada prioridade ao planeamento de estratégias para melhorar a retenção de enfermeiros, reduzir o número de pacientes por enfermeiro e promover bons ambientes de trabalho nos hospitais.

## Introduction

Job satisfaction is a construct difficult to define that involves cognitive and emotional aspects^1^ and which expresses the perceptions people have of their work.^2^^,^^3^ The degree to which the work environment fulfills the expectations of the individuals is that which defines the level of satisfaction.^2^ The job satisfaction of health teams is of much relevance, given that it is associated with better productivity and quality of work and it is a primary factor in the individual’s well-being.^4^^,^^5^ Job dissatisfaction, in turn, is a factor, among others, associated with absenteeism, rotation, deficient work results, dehumanization of care.^4^^-^^6^ International studies indicate some determinants of job satisfaction in nursing professionals.^5^^,^^7^ Most of the studies have analyzed two categories of determinants. In the first place, there are the personal characteristics of the nurses, like age, sex, education, years of experience, among others.^3^ In the second place, a multiplicity of organizational factors has been studied, like work environment, availability of resources, assignments, the leadership style of heads, or recognition.^3^^,^^5^^,^^7^

In Chile, recent studies account for the profiles of nurses working in public and private sector hospitals and for some of the characteristics of their work environments.^8^^-^^10^ The evidence gathered shows that there are factors of the nurses and of the hospital setting that could be impacting negatively upon the satisfaction of these professionals. Among the personal factors, the age of the nurses is highlighted. Some studies indicate that young nurses tend to be less satisfied than those who are older and, in Chile, an important number of nurses employed in hospitals does not reach 30 years of age.^10^ Regarding factors of the environment, it is known that assignments of nurses in Chilean hospitals are insufficient,^10^ which generates work overload and lack of time to perform all the care tasks.^11^^-^^13^ However, few studies have evaluated the job satisfaction of nurses and those that have are old and show specific realities from two Chilean regions.

Two decades ago, the level of satisfaction of nurses from Region VIII of Chile was measured, analyzing some determinant factors and the existence of differences between those working in private and public hospitals. Among their findings, these reported low overall level of satisfaction among the nurses and worse results in nurses from public hospitals.^14^ A subsequent study, carried out in Santiago de Chile, whose aim was to measure job satisfaction among nurses from five hospitals and the association of satisfaction with the leadership styles of the heads, established that nearly 42% of the nurses was not very satisfied or dissatisfied with their job.^15^ Both studies are now rather old and present regional realities not necessarily generalizable to the entire country. 

Within the international perspective, a recent meta-analysis demonstrated that nurses in better work environments have between 28% and 32% less probabilities of job dissatisfaction.^16^ Countries, like the Czech Republic, have declared that a positive work environment has a favorable impact on the nurses’ job satisfaction.^17^ In Brazil, the results are similar. ^18^ In Taiwan, less lower levels of satisfaction were found among nurses, evidencing that greater support to autonomy was associated positively with job satisfaction, as well as the interventions to favor empowerment and diminish burnout.^19^

The purpose of this research was to measure, at the national scope, the satisfaction of Chilean nurses working in public and private hospitals, and establish personal and institutional determinants associated with satisfaction. This study derives from the project RN4CAST-Chile (Linda H. Aiken, University of Pennsylvania, principal researcher) whose purpose was to understand the contribution by nurses to the results of patients and which factors of the work environment affect the nurses’ performance and wellbeing. The RN4CAST protocol is described in greater detail in other publications.^20^^,^^21^ This study was led by the Schools of Nursing at the University of Pennsylvania, in the United States, and at Universidad de los Andes in Chile. It had collaboration from another three Chilean universities: Universidad Católica del Maule, Pontificia Universidad Católica de Chile, and Universidad de La Serena. The study was conducted between 2016 and 2019, which is why the results presented are the reflection of the nurses’ situation prior to the start of the COVID-19 pandemic.

## Methods

An observational, cross-section study was conducted among nurses in medical, surgical, or medical-surgical units from high-complexity general hospitals throughout the Chilean territory. In the first instance, the hospitals were selected. Within the context of the global project, it was necessary for the hospitals selected to have implemented the codification of hospital discharges with system of Groups Related to Diagnosis. This criterion limited to 45 the universe of high-complexity general hospitals to study; 37 public and 8 private hospitals. 

After defining the hospitals, the nurses were selected from medical, surgical, or medical-surgical units. Besides the unit, the only selection criterion was that they had to nurses in charge of direct patient care. Thereby, nurses in management tasks were excluded. The study did not perform a sampling, rather, it invited to participate all nurses who met the inclusion criteria. To know the total number (*n* = 2,173), prior to collecting data, a census was made through the hospital’s head nurse. Knowledge of the total number of nurses of the population permitted calculating the participation rate. The nurses participating in the study had to answer a self-administered survey with questions about their work environment, number of patients assigned to their care, and about some job indicators among which was included satisfaction. In addition, questions were included of sociodemographic nature and about education and professional experience of the nurses. The survey was answered on paper, with previous signed informed consent. The consent was signed in two copies, one for the nurse participating and another for the research team. Data collection lasted 18 months, taking place between May 2017 and October 2018.

The variables of interest were job satisfaction and its possible determinants. Among these, personal determinants (demographic, training, and professional experience) and institutional determinants were explored, which included the organizational and structural aspects of the hospitals.

*Job satisfaction*: was measured through a single question with four possible responses in an ordinal measurement scale: “very satisfied”, “somewhat satisfied”, “somewhat dissatisfied”, “very dissatisfied”. For the statistical analyses, the variable was dichotomized, separating nurses who reported being very satisfied with their job from those who stated something else. Further, a satisfaction evaluation was added with respect to specific work aspects (salary, training opportunities, among others). These questions, measured with the same Likert scale, were managed in the same way as the satisfaction variable. 

*Personal determinants*: sex was measured as a dichotomous qualitative variable (woman-man). Age, years of professional experience, and years working in the current hospital were measured as continuous variables expressed in years. Specialization training and graduate degree was measured through the following question: “*What is the highest level of postgraduate teaching you have achieved?*” The possible responses included “*none*”, “*specialization*”, “*master’s*”, and “*doctorate*”. Thereafter, this variable was dichotomized to group the nurses who answered “none” in one category and all the rest in the other, representing those who do have post-graduate studies or graduate degree. 

*Institutional determinants*: first, some variables of organizational nature were studied. One of them was the quality of the work environment; the *Practice Environment Scale of the Nursing Work Index* -PES-NWI-^22^ was used to measure this. The instrument, which is the most widely used globally, is validated in Spanish.^23^ It comprises 32 questions that assess five dimensions of the nursing work environment: participation by nurses in decision making within the hospital, institutional commitment with quality, communication and leadership from management, availability of sufficient resources including those of personnel, and communication between physicians and nurses. Each question is answered with a Likert scale of four categories between “*totally disagree*” and “*totally agree*”, with values from 1 to 4. From the questions corresponding to each of the five subdimensions, an average score was obtained by each hospital. Then, these five values were averaged to obtain a final average that expresses the quality of the work environment in each hospital. This average was measured as a continuous variable with values from 1 to 4. Together with the work environment, staff assignments were measured expressed as assignment of patients per nurses. To measure this variable, each nurse was asked how many patients and nurses were there in their unit during the last work shift. Staff assignment was calculated by dividing the patients by the number of nurses. From the nurses working in the same hospital, an average value was obtained of patients per nurse in said hospital. The structural variables of the hospitals were also measured. Among these were included the hospital property (public or private), size expressed in number of beds, and location (differentiating hospitals in the capital, Santiago, from those in other cities). 

For the data analysis, firstly, participation rates were calculated for both hospitals and nurses. Next, the distribution of the quantitative variables was evaluated by applying the Kolmogorov-Smirnov test to identify variables of normal and non-normal distribution. The characteristics of the nurses and hospitals were described. For the qualitative variables, frequencies and percentages were used and, for the quantitative variables, means and standard deviations were used if the variable had normal distribution, and median and interquartile range if the variable did not have normal distribution. A descriptive analysis was performed of the nurses’ overall satisfaction and in relation with specific aspects of their work. Satisfaction prevalence, expressed in percentage, was measured by dividing the number of nurses who stated being very satisfied with their current work by the number of nurses who participated in the study and multiplying by one hundred. Then, the characteristics of the nurses and hospitals were contrasted between the nurses who said they were very satisfied with their job and all the rest. To evaluate if the differences were significant, the study applied Chi-squared tests in the case of categorical variables and Student’s t or Mann-Whitney U tests for continuous variables, according to their distribution, with a significance level of α = 0.05. 

To establish the determinant factors of satisfaction among nurses, logistic regression models were used with the dichotomic variable of satisfaction as result variable (very satisfied nurses (1) and not very satisfied nurses (0)). The selection of possible determinants was made primarily based on the evidence already existing in the literature, seeking to avoid multicollinearity among some variables. The possible determinants were grouped, according to their type, into three blocks and a hierarchical logistic regression analysis was applied, following a nested models design. The simplest model was constructed with the nurses’ personal characteristics. The second model incorporated the organizational characteristics of the hospitals. Lastly, the third model incorporated structural characteristics of the hospitals. To evaluate the goodness of fit of the models, Akaike and Bayesian (AIC and BIC) criteria were applied. Analyses were carried out with the STATA 17.0 BE program. 

This study was approved by the Scientific Ethics Committee at Universidad de los Andes, Chile (CEC201613). Some local committees, whether from the local divisions of the Ministry of Health or from the very hospitals, also reviewed the Project for approval. The study presented no risks to the participants. Participation by the nurses was voluntary and anonymous, with prior signed informed consent. 

During the execution of the study, the ethical principles of respect, beneficence, and justice were safeguarded. The dignity of the individuals was respected by inviting them to participate voluntarily and without coercion. Beneficence was protected by means of anonymity, to assure participants that the information collected could not be used against them by their heads or people in higher positions. Justice was lived in terms of non-discrimination of the possible participants, given that all those who met the inclusion criteria were invited to participate. 

## Results

The study had 89% participation rate among the hospitals (40/45) and 75% among the nurses (1,632/2,173). Two of the missing hospitals did not wish to participate in the study due to circumstantial internal reasons and the other three communicated their authorization to the research team once the data collection process had been closed. Most of the participating hospitals were public, and the distribution among the country’s northern zone, southern zone, and Santiago was quite homogeneous and coherent with the population distribution (25%, 40%, and 35%, respectively). The assignment average in hospitals was 14 patients per nurse. 

The nurses studied were mostly women and nearly 60% of the participants was 30 years old or less and had five or less years of professional experience; 27% had post-graduate studies or graduate degree, mostly clinical specializations and, in isolated cases, Master’s studies. Table 1 describes the principal characteristics of the hospitals and nurses. 

**Table 1:** General characteristics of 1,632 nurses who work in 40 Chilean hospitals

Nurses	Valor
Men; *n* (%)	190 (11.7)
Age; median (range)	30.0 (20.0 - 66.0)
Years of professional experience; median (range)	4.0 (0 - 45.0)
With specialization or graduate degree; *n* (%)	440 (27.3)
Hospitals	
Public; *n* (%)	34 (85.0)
Located in Santiago; *n* (%)	16 (40.0)
Size; mean (SD)	450 (145)
Patients per nurse; median (SD)	13.0 (5.9 - 23.0)
Score of work environment; mean (SD)	2.6 (0.2)

Table 2 presents descriptive data on the number and percentage of nurses who state being very satisfied with their current work. One in every five nurses, approximately, is very satisfied. With respect to specific work aspects, there is a very low percentage of nurses who report being satisfied with growth and professional formation opportunities. Moreover, over 25% of the nurses indicate being very satisfied with their professional autonomy. 

**Table 2:** Proportion of nurses “very satisfied” with their work according to total and specific aspects of such (*n* = 1,632)

Satisfaction	***n* (%)**
Overall satisfaction	342 (21.0)
Satisfaction with specific work aspects:	
Training opportunities	164 (10.0)
Professional growth opportunities	183 (11.2)
Salary	271 (16.6)
Vacations	292 (17.9)
Professional status	336 (20.6)
Work shift	382 (23.4)
Medical leaves	420 (25.7)
Autonomy	433 (26.5)

Table 3 contrasts differences in personal and institutional characteristics among nurses who reported being very satisfied with their job and those whose degree of satisfaction was lower. Between the group of very satisfied nurses and that of nurses who were less satisfied, no significant differences exist in the proportion of women, age, and years of professional experience or of permanency in the institution, or in the proportion of nurses with postgraduate training. Significant differences do exist between both groups with respect to the characteristics of the institutions in which they work. A lower percentage of very satisfied nurses works in public hospitals, compared with those less satisfied, has lower workloads, and works in hospitals with better quality of work environment. All the subdimensions of the work environment show a better score among the very satisfied nurses compared with those from the group that is not very satisfied (these scores are not shown in the table). 

**Table 3:** Differences in personal characteristics and characteristics related to work between very satisfied nurses and those not very satisfied (*n* = 1,632)

	**Very satisfied *n* = 342**	**Not very satisfied *n* = 1,290**	*p-value*
Personal characteristics			
Men; *n* (%)	49 (14.3)	150 (11.0)	0.090
Age; median (range)	29 (20 - 66)	30 (22 - 63)	0.279
Years of professional experience; median (range)	4.0 (0 - 45)	4.0 (0 - 41)	0.002
With postgraduate training or graduate degree; *n* (%)	91 (26.7)	349 (27.5)	0.081
Characteristics related to work			
Nurses from public hospitals; *n* (%)	280 (81.8)	1.115 (86.4)	0.033
Nurses in hospitals of Santiago; *n* (%)	157 (45.9)	603 (46.7)	0.782
Patients per nurse; median (range)	12.5 (5.9 - 23.0)	13.0 (5.9 - 23.0)	0.006
Quality of the work environment; mean (SD)	2.66 (0.19)	2.58 (0.20)	< 0.0001

Three models were constructed to evaluate the determinants of nurses’ job satisfaction. Model 1, which incorporated personal and formation characteristics of the nurses, did not yield any statistically significant result; although in some, the size of the effect measured for the sex variable sex, for example, results rather large (OR: 1.39). Model 2 integrated two variables related with the organization of the hospitals: assignments and work environment. This model resulted significantly better in terms of explaining the factors associated with the nurses’ satisfaction. In this new model, significant association appears among satisfaction and the nurses’ sex and age. Being a male is associated with nearly 50% greater probability of being satisfied with the job work and for every five years of increase in age of the nurses, there is almost 10% more likelihood of being satisfied. Together with the nurses’ characteristics, model 2 shows that both organizational variables analyzed are associated significantly with the nurses’ satisfaction, with the effect of the work environment being particularly important. For every 1 standard deviation increase in the average score of hospital work environment, the likelihood of the nurses being satisfied increases by almost 40% (values are shown in Table 4). 

**Table 4:** Hierarchical logistic regression analysis of the association among nurses’ characteristics, hospital characteristics, and job satisfaction (*n* = 1,632)

Variables	Model 1				Model 2		
	OR	95% CI	***p*-value**		OR	95% CI	***p-*value**
Sex (male)	1.36	0.95 - 1.92	0.089		1.49	1.04 - 2.12	0.029
Age †	1.01	0.98 - 1.16	0.121		1.09	1.01 - 1.18	0.035
Graduate studies	0.92	0.70 - 1.21	0.547		0.89	0.67 - 1.18	0.425
Patients per nurse					0.96	0.93 - 0.99	0.049
Work environment‡					1.39	1.23 - 1.57	< 0.0001
† The age variable was operationalized so that the effect presented corresponds to that associated with 5-year increments. ‡ The work environment variable is standardized so that the effect presented corresponds to increments of 1 standard deviation in the environment score.					

A third model was evaluated, which added a new block of determinants that corresponded to the structural characteristics of the hospitals, like the condition of being public or private or their location. The inclusion of these variables showed no significant differences with respect to model 2, which is why the results from model 3 are not presented. 

## Discussion

This study is the first of national scale that measures nurses’ satisfaction in Chilean hospitals. Its results reveal that only one in every five nurses is very satisfied with their job. In specific aspects of satisfaction, only one in every ten nurses is very satisfied with respect to training opportunities and professional growth. This finding agrees with results reported 20 years ago, which indicated that professional promotions were among the important factors of dissatisfaction of Chilean nurses.^14^ In effect, the professional career of nurses from hospitals has very few incentives; currently, work is being done in the Ministry of Health on a policy that grants formal recognition to the postgraduate training of nurses, specially to those with clinical specializations, and to the development of advanced nursing practice roles. In some European countries, like Slovenia, the postgraduate training of nurses is an incipient reality, a fact that impacts negatively on their job satisfaction. ^(^^24^ In other countries, like Switzerland, more progress has been made in this line, positively impacting satisfaction.^25^ In the United States, postgraduate training is widespread, which favors the nurses’ job satisfaction.^26^^,^^27^

In the bivariate analysis, very satisfied nurses, compared with those that are not very satisfied, were not differentiated significantly regarding their demographic characteristics of professional experience. However, in the multivariate analysis that accounted for the determinants of satisfaction, an effect of the sex and age variables was seen on the condition of being very satisfied with the job. Male nurses, and those who were older, showed significantly higher probability of being very satisfied with their job than women or the younger male nurses. Studies in other countries present inconsistent results with respect to the role of sex and age on nurses’ satisfaction.^3^^,^^28^. Within the context of Chilean hospitals, with mostly young nurses, it makes sense to think that increased age is associated with greater satisfaction. The few older nurses very likely have remained in their institutions because of being more satisfied. Years of professional experience were not included in the predictive models to avoid multicollinearity problems with the age variable, but it is possible that years of experience are a factor positively associated with satisfaction, as shown by other studies.^28^

Highlighted, among the results of the present study, is the role of the work environment as strong predictor of nurses’ satisfaction, even above other organizational factors, like staff assignments. The results are coherent with those from a recent meta-analysis.^29^ The aggregate analysis regarding the effect of the environment on nurses' satisfaction, considering the opposite of dissatisfaction, showed that nurses in hospitals with a good environment are 32% less likely to be dissatisfied with their job.^29^ This is an effect size very similar to that found in this study. There are studies that delve into the specific environmental factors that may be more closely associated with satisfaction.^28^ For example, some of the factors described are nurses’ perception of lack of support from their coworkers, the salary, autonomy, among others. In this study, although the scale to measure the quality of the work environment has five subdimensions, the analysis was performed with the global measurement of the quality of the environment, which is the form originally described to use this instrument.^22^ Thus, a field remains open for future research with respect to the specific work environment aspects that impact more critically on the satisfaction of Chilean nurses. 

Staff assignments were also associated with nurses’ satisfaction. The higher the patient load, the lower the probability that nurses will be satisfied. The evidence already accumulated for two decades on the effect of staff assignments on nurses’ job indicators, among them satisfaction, is quite robust and coherent with the results of this study.^30^^,^^31^ However, it must be highlighted that, within the Chilean hospital context, in which the average load of patients per nurse is much higher than that described in other countries;^30^ this result should be taken into special consideration. 

The structural variables of the hospitals had no role as predictors of nurses’ satisfaction. Contrary to what may have been thought, the institutional characteristic of public or private hospital was not associated with nurses’ job satisfaction. This differs from the previous studies conducted in our country that indicated that nurses from the private area had higher levels of satisfaction than those from the public area.^14^ Two possible explanations exist. On the one hand, it may be that hospitals, independent of belonging to the public or private network of health providers, do not differ in terms of creating work environments that are more conducive to satisfaction. Furthermore, in this study a low proportion of hospitals was private, which may have limited statistical power to investigate significant differences between public and private hospitals. 

The study, herein, had some limitations. Its cross-sectional design limits the capacity to establish causal relationships among the variables of interest. Also, the study only included nurses from medical, surgical, or medical-surgical units; hence, caution is required when generalizing these results to other types of hospital units. However, the units studied represent nearly 50% of the beds in each of the hospitals, thus, we believe that the results probably capture rather well the hospital reality as a whole. 

In conclusion, the findings obtained account for only one in every five nurses in medical-surgical units of hospitals from the public and private network throughout the Chilean territory being very satisfied with their job. The determinants factors associated with nurses’ personal characteristics are not modifiable, but work can be done by identifying the points that generate differences between the satisfaction of male and female nurses. Policies can also be implemented that favor the retention of older nurses and which, when being more satisfied, promote positive organizational climates. Staff assignments and the work environment are determinants of satisfaction among Chilean nurses. Improving staff assignments is a permanent challenge, but it comes at a high cost that may be difficult to assume in some hospitals, while the creation of good-quality work environments is a challenge at the reach of all. 

This study contributes to the nursing discipline by delivering new knowledge that contributes to the management setting of health institutions. Bearing in mind the evidence gathered, nurses dedicated to management, especially those in management or directive positions, should include in their strategic plans an objective seeking to improve the satisfaction of the nursing staff. Among the actions leading to said objective, priority should be given to strategies of retention, improved staff assignments, and promotion of good work environments. 
